# Physical activity screening to recruit inactive randomized controlled trial participants: how much is too much?

**DOI:** 10.1186/s13063-015-0976-7

**Published:** 2015-10-08

**Authors:** Corneel Vandelanotte, Robert Stanton, Amanda L. Rebar, Anetta K. Van Itallie, Cristina M. Caperchione, Mitch J. Duncan, Trevor N. Savage, Richard R. Rosenkranz, Gregory S. Kolt

**Affiliations:** Physical Activity Research Group, School for Human Health and Social Science, Central Queensland University, Rockhampton, QLD Australia; School for Medical and Applied Sciences, Central Queensland University, Rockhampton, QLD Australia; School of Health and Exercise Sciences, University of British Columbia, Kelowna, BC Canada; Priority Research Centre for Physical Activity and Nutrition, Faculty of Health and Medicine, School of Medicine and Public Health, University of Newcastle, Newcastle, NSW Australia; School of Science and Health, Western Sydney University, Sydney, NSW Australia; Department of Human Nutrition, Kansas State University, Manhattan, KS USA

**Keywords:** measurement, physical activity, randomized controlled trial, recruitment, screening, single-item, study design, study protocol

## Abstract

**Abstract:**

Screening physical activity levels is common in trials to increase physical activity in inactive populations. Commonly applied single-item screening tools might not always be effective in identifying those who are inactive. We applied the more extensive Active Australia Survey to identify inactive people among those who had initially been misclassified as too active using a single-item measure. Those enrolled after the Active Australia Survey screening had significantly higher physical activity levels at subsequent baseline assessment. Thus, more extensive screening measures might result in the inclusion of participants who would otherwise be excluded, possibly introducing unwanted bias.

**Trial registration:**

Australian New Zealand Clinical Trials Registry, ACTRN12611000157976.

## Findings

Long-term physical inactivity is associated with increased levels of chronic disease and reduced quality of life [1]. The majority of the population, however, does not engage in enough physical activity to gain optimum health benefits [2]. Randomized controlled trials are being conducted, to examine the effectiveness of interventions to increase physical activity. Many of these trials screen physical activity levels of potential study participants with the aim of only recruiting participants who are low-active [3].

Pre-randomization screening to enroll low-active participants might increase intervention effect sizes; which might necessitate a smaller study sample to demonstrate between-group differences [3]. People already meeting physical activity recommendations are often attracted to physical activity interventions, though they are typically not the target audience for these kinds of intervention, as there are fewer public health benefits in increasing activity in active people, especially when resources are limited [4]. Participants with low activity levels also have a greater capacity to increase activity levels and ceiling effects are less likely to occur; thus, the likelihood that the intervention will be successful in demonstrating its effectiveness is greater [5]. Finally, active people are known to be responsive to physical activity messages, even in the absence of an intervention to help them become more active. Therefore, to decrease the probability of the control group becoming more active (which would undermine study outcomes) recruiting a sample that is resistant to change in the absence of an intervention (i.e., inactive people) is beneficial [6].

Waters *et al.* [3] indicated that 18 out of 23 trials that they evaluated implemented some sort of physical activity screening, but that the methods applied and the physical activity cut-off points used to determine eligibility varied greatly. The majority of studies have used single-item physical activity screening tools, mainly to ensure that participant recruitment is efficient and of little burden to those wanting to participate [7]. Although some studies have demonstrated acceptable validity for single-item measures [7], it has also been commonly reported that large proportions of participants still meet physical activity guidelines at the trial baseline assessment when more rigorous physical activity measures are applied, such as full-length questionnaires or objective accelerometer-based measures [8].

In this study, we examined the effectiveness of a two-stage physical activity screening process as part of a three-arm randomized controlled trial (‘Walk 2.0’) [9]. The Walk 2.0 trial investigated the effectiveness of two web-based physical activity interventions (a traditional web 1.0 intervention and an intervention including online social networking and other web 2.0 components) compared with a print-based intervention [9]. A total of 1244 people completed the initial screening questionnaire and 504 people were randomized into one of three groups. People were excluded based on a number of screening criteria (e.g., under 18 years of age, no internet access, medical condition that prevents increased activity), although the numbers presented here only relate to the outcomes of the physical activity screening. A full overview of why and at what stage participants were excluded from the trial will be presented elsewhere.

Physical activity screening was applied in two steps. The first step included application of a commonly used single-item physical activity measure: ‘As a rule, do you engage in at least half an hour of moderate or vigorous exercise (such as walking or a sport) on five or more days of the week (yes/no)?’ [10]. This measure has demonstrated good concurrent validity, identifying 77 % of those who were physically inactive according to a more extensive assessment of physical activity [11]. Those who answered ‘no’ to this single-item measure were deemed eligible to participate; however, those who answered ‘yes’ underwent a second step of telephone-administered screening, using the Active Australia Survey [12]. This survey includes items to assess the duration and frequency of walking, and of moderate and vigorous physical activity in the previous week and has demonstrated acceptable reliability and validity [13]. Participants were only eligible to participate when reporting less than 150 min/week of physical activity using this survey. This two-step protocol was applied to minimize participant burden (for those who were eligible after the first step), and maximize the use of resources allocated to recruitment (to ensure that eligible participants were not excluded if the initial assessment was inaccurate).

A total of 418 people answered ‘no’ to the single-item measure and were accepted into the trial, whereas the 370 people who answered ‘yes’ underwent further screening using the Active Australia Survey. Of these, 284 (76.8 %) reported more than 150 min/week of physical activity and were excluded as too active to participate in the trial. The average level of physical activity of those excluded was 306 ± 217 min/week. The 86 (23.2 %) people who were included reported an average of 80 ± 48 min/week of physical activity at the time of screening.

When examining baseline physical activity levels (see Table 1), we observed not only that a large number of participants were exceeding 150 min/week of physical activity regardless of screening protocol, but also that those who had participated in the two-step screening process were significantly more physically active at baseline assessment (measured both subjectively and objectively (Actigraph accelerometry)), compared with those who had only undergone the single-item screening. While some test values only trended toward significance (*P* = 0.05–0.10), the differences between groups seem to be mainly driven by differences in walking levels.

**Table 1:** Baseline physical activity for participants screened with a single-item only (first step) and for those screened more extensively (second step)

Baseline physical activity outcomes	First step screening, (*n* = 418)	Second step screening (*n* = 86)	*t* or χ^2^ test	*P*
Active Australia Survey:				
Walking (min/week)	119 ± 149	162 ± 168	2.37	0.018
Moderate intensity (min/week)	38 ± 97	27 ± 63	−1.02	0.307
Vigorous intensity (min/week)	46 ± 95	44 ± 85	−0.15	0.881
Walking + moderate + vigorous intensity (min/week)	204 ± 247	234 ± 216	1.04	0.297
Achieves 150 min/week of moderate + vigorous intensity (%)	41.2	55.8	6.14	0.013
Actigraph Accelerometry:				
Steps (number/day)	7057 ± 2314	8166 ± 2727	3.41	0.001
Moderate intensity (min/week)	157 ± 118	190 ± 140	2.61	0.031
Vigorous intensity (min/week)	4.9 ± 21.3	2.3 ± 7.3	−1.89	0.059
Moderate + vigorous intensity (min/week)	162 ± 141	192 ± 141	1.91	0.057
Achieves 150 min/week of moderate + vigorous intensity (%)	43.6	50.6	1.33	0.249

An important point to note is that neither screening approach (single-step or dual-step) was particularly effective in preventing people who were too active from being enrolled in the study; and that screening using objective measures (accelerometry)—despite being burdensome, time intensively, and costly—may thus be preferable, as fewer active participants would subsequently be enrolled. Further, it is challenging to explain the counterintuitive differences with regards of single- and dual-step screening outcomes. One plausible explanation may be related to social desirability bias [14]. That is, some participants may have sensed their likelihood of inclusion in the randomized controlled trial would be higher if reporting a lower level of physical activity on the subsequent screening measure. Another explanation may be that the more extensive screening increased physical activity awareness [3], resulting in more efforts to be active prior to the baseline assessment. These findings highlight that recruitment procedures need to be designed carefully, as they might introduce potentially unwanted and unexpected biases.
